# Preliminary identification of clinical cut-off of the vegetarian vegan eating disorder screener (V-EDS) in a community and self-reported clinical sample of vegetarians and vegans

**DOI:** 10.1186/s40337-024-00969-2

**Published:** 2024-01-24

**Authors:** Courtney P. McLean, Zhibin Chen, Joanne Fielding, Gemma Sharp

**Affiliations:** Department of Neuroscience, Monash University, 99 Commercial Rd, Melbourne, VIC Australia

**Keywords:** V-EDS, Eating disorder, Vegetarian, Vegan, Cut-off score, ROC curve analysis, Assessment

## Abstract

**Background:**

The vegetarian vegan eating disorder screener (V-EDS) is an 18-item self-report screening tool designed to assess the unique elements of eating disorder symptomology in vegetarians and vegans. Previous results have suggested strong initial psychometric properties in non-clinical community samples of vegetarians and vegans. The present study sought to identify a preliminary threshold cut-off score to discriminate eating disorder pathology in a self-reported clinical and community sample.

**Methods:**

This study involved secondary analysis using data collected in McLean et al. (Development and preliminary validation of a novel eating disorder screening tool for vegetarians and vegans: the V-EDS, 2023), comprising 599 non-clinical participants and 51 self-reported clinical participants. Receiver operating characteristic (ROC) curve analysis was used to compute possible cut-off values for the V-EDS. Results: ROC analysis indicated good performance of the V-EDS (area under the curve = 0.87), with integration of the Youden index demonstrating a global score of ≥ 18 to be optimal in predicting clinical caseness with good sensitivity (0.804) and specificity (0.843).

**Conclusions:**

The present study fills an important gap as the first to investigate an optimal V-EDS score to discriminate level of impairment from eating disorder pathology in a sample of vegetarian and vegan community and self-reported clinical participants. We extend the utility of the V-EDS in discovering good discrimination power in classifying clinical caseness with a cut-off score of 18 shown to optimise the trade-off between sensitivity and specificity. Future research should focus on expanding the psychometric properties of the V-EDS in larger and more diverse participant groups, including gender, age, cultural identity, and eating disorder history.

## Abstract (plain language summary)

This study builds on the preliminary validation of a novel eating disorder screening tool for people adhering to a vegetarian and vegan diet called the V-EDS. In this study, we set out to develop a cut-off score for the V-EDS to distinguish people needing further evaluation for a possible eating disorder within the community. We found a global V-EDS score of ≥ 18 to be ideal in distinguishing between eating disorder symptomatic and non-eating disorder groups. In future, the V-EDS may prove useful for initial screening and symptom progression of eating disorders across both clinical and research settings.

## Introduction

The uptake of vegetarianism and veganism is on the rise and is thought to be driven by various factors, including animal welfare and environmental sustainability concerns and positive health benefits [1, 2]. It is widely recognised within the eating disorder field that vegetarianism and veganism may be related to an increased risk of eating disorder pathology [3–5]. For example, some people may be attracted to vegetarianism or veganism to provide a socially acceptable way to justify the exclusion of food groups and disguise disordered eating [6, 7]. Though it is well established that vegetarians and vegans have important eating attitudes and behaviours that are uniquely different from omnivores [8], the generalisability of eating disorder assessment tools is often assumed in these populations [9]. It has been noted that eating disorder tools may be inappropriate to use in vegetarians and vegans for several reasons. For example, such measures may be unable to decipher motivations for dietary restraint, in turn, misidentifying the restraint required to follow a vegetarian or vegan diet with the restraint required to restrict food to change body weight or shape [8]. Consequently, it remains important to employ specialised screening tools to differentiate vegetarian and vegan eating behaviours from pathological eating behaviours, in turn, allowing for the identification of those requiring further intervention.

A newly developed screening tool that aims to overcome challenges in assessing eating disorder pathology in the growing population of vegetarians and vegans is the vegetarian vegan eating disorder screener (V-EDS; [2, 10]). The V-EDS is a relatively brief 18-item self-report instrument designed to assess symptoms of eating disorders in vegetarians and vegans over the past seven days. Developed across four phases of construct and item development, the V-EDS relied on the personal experiences of vegetarians, vegans, and people with lived eating disorder experience, and the professional expertise of psychologists and dietitians working in the eating disorder field. The V-EDS was designed to be integrated into research and clinical settings to discriminate between fundamental factors driving increasing eating disorder pathology (e.g., restricting food to influence weight versus restricting food groups to follow a vegetarian or vegan diet). The V-EDS also attempts to overcome some limitations in the field as a quick, inexpensive, and efficient way to detect those who may need further evaluation [11, 12]. The V-EDS supports a unidimensional factor structure with excellent internal consistency (α = 0.95–0.96) and convergent validity (0.87–0.88), and moderate discriminate validity (0.45–0.55) in separate samples of vegetarians and vegans [10]. Though the tool is yet to adopt an optimal cut-off value to distinguish between respondents requiring further evaluation. Identifying a clinical cut-off value has the potential to enhance the clinical utility and applicability of the V-EDS [13, 14], in turn, improving the accessibility of vegetarian and vegan-identifying patients to specialised eating disorder treatment. Therefore, this study aims to identify a V-EDS cut-off score that optimises both sensitivity and specificity to discriminate eating disorder pathology in a self-reported clinical and community sample of vegetarians and vegans.

## Methods

This study involved secondary analysis of data collected for the development and validation of the V-EDS described in detail in McLean et al. [10]. Ethics approval was obtained from the Monash University Human Research Ethics Committee (Project ID: 30651) and participants were informed of the purpose of the study and provided informed consent prior to participating.

### Participants

Participants were advertised through previous participant databases, social media advertisements (e.g., Vegans in Australia Facebook group), and eating disorder charity networks to complete an online survey on vegetarian and vegan eating behaviours and attitudes. Participants were required to be 18 years or over, residing in Australia, and adhering to a vegetarian or vegan diet to be eligible. Participants who identified as “meat-reducers” (i.e., flexitarians, semi-vegetarians, pescatarians) were excluded from the study owing to our specific focus on vegetarian and vegan eating behaviours.

### Measures

*Participant demographic characteristics* Participants responded to several demographic characteristic questions (e.g., age, gender, ethnicity, religion, education level) and specific questions about their dietary adherence including duration of adherence and motivations.

Vegetarian vegan eating disorder screener (V-EDS) [10]*.* The V-EDS is an 18-item self-report measure designed to assess symptoms of eating disorder pathology in vegetarians and vegans over the past seven days. The V-EDS comprises six dietary characteristic items and 12 behavioural and attitudinal items. The dietary characteristic items can be used to provide clinical information around the respondent’s dietary attitudes (e.g., *“Your vegetarian/vegan diet is a part of your identity”*) and are along a 5-point Likert scale from s*trongly disagree* to *strongly agree*. The following behavioural and attitudinal items (e.g., *“Has the way you thought about food become intrusive?”*) measure the presence of eating disorder pathology, rated along a 5-point Likert scale from *no days* to *every day*. The V-EDS has been found to support a unidimensional factor structure with strong initial internal consistency (α = 0.95–0.96) and convergent validity (0.87–0.88), and moderate discriminate validity (0.45–0.55). Internal consistency in the present study was excellent (α = 0.95, ω = 0.95).

### Procedure

Briefly, participants were advertised with a link to the online survey and responded to demographic characteristic questions, including age, gender, ethnicity, religion, and highest completed education, followed by the V-EDS. Participants self-reported their eating disorder diagnosis if applicable.

### Statistical analysis

Statistical analyses were performed using SPSS Version 27 [15]. Descriptive statistics were calculated, including means and percentages for participant characteristics (age, gender, dietary status) and medians for V-EDS scores, and presented by study group (clinical, non-clinical). Differences in participant characteristics by group were examined using *t*-tests for continuous variables (age) and chi-square tests for categorical variables (gender, dietary status). Receiver operating characteristic (ROC) curve analysis was used to compute possible cut-off values for the V-EDS. The ROC is a plot that displays the trade-off between the sensitivity and its 1-specificity across a range of threshold values. The area under the curve (AUC) was calculated to provide an overall test performance statistic based on the trapezoidal rule. The discrimination power of the V-EDS is interpreted according to the AUC as non-informative (AUC = 0.50), poor (0.50 < AUC < 0.70), good (0.70 < AUC < 0.90), excellent (0.90 < AUC < 1.00), and perfect (AUC = 1.00; [16]). For this study, the optimal cut-off was identified using the Youden index which puts equal weight on sensitivity and specificity (sensitivity + sensitivity − 1), resulting in an index score between 0 and 1 [17, 18]. The cut-off point for having an acceptance Youden index is 50%, with higher scores indicating better performance. Statistical significance level was set at *p* < 0.05.

## Results

The total sample consisted of 650 vegetarian and vegan participants, including 599 non-clinical participants (mean age [*M*]: 34.72 years, standard deviation [*SD*] = 11.06; 504 [84%] females, 221 [37%] vegetarians) and 51 self-reported eating disorder participants (i.e., “clinical” participants; mean age: 29.41 years, *SD* = 9.65, 47 [92%] female, 24 [47%] vegetarian). There were significant differences in age between participants with the non-clinical participants being significantly older (*t*(648) = − 3.32, *p* < 0.001) than the clinical participants, but not gender (*χ*^*2*^[3] = 8.30, *p* = 0.081) or dietary status (*χ*^*2*^[10] = 2.07, *p* = 0.15). Of the 51 clinical participants, most self-reported a current diagnosis of anorexia nervosa (*n* = 24, 47%), followed by binge eating disorder (*n* = 7, 14%), atypical anorexia nervosa (*n* = 5, 10%), other specified feeding and eating disorder (OSFED; *n* = 4, 8%;), bulimia nervosa (*n* = 3, 6%), and eating disorder not otherwise specified (ENDOS; *n* = 2, 4%). Six participants preferred not to self-classify their eating disorder diagnosis.

Median V-EDS scores were 4.00 (interquartile range [*IQR*]: 1.00–14.00, *M* = 9.47, *SD* = 11.78) for the total sample, with a median of 4.00 (*IQR*: 1.00–11.00, *M* = 7.86, *SD* = 10.01) for the non-clinical community sample and 28.00 (*IQR*: 18.00–42.00, *M* = 28.41, *SD* = 14.38) for the self-reported clinical sample. An independent samples *t*-test found participants within the clinical sample had statistically significantly higher V-EDS scores compared to participants within the community sample, *t*(648)  = 13.53, *p* < 0.001. Table 1 outlines the mean, standard deviation, median, interquartile range, and group differences of V-EDS scores across vegetarian, vegan, and clinical sample groups.

**Table 1:** V-EDS scores across vegetarian, vegan, and clinical sample subgroups

	*M (SD)*	*Mdn (IQR)*	Test statistic
Vegetarian (*n* = 221)	8.67 (10.46)	4.00 (11.00)	*t*(597) = 1.52, *p* = 0.129
Vegan (*n* = 378)	7.39 (9.72)	3.00 (10.00)	
Vegetarian with lived eating disorder experience (*n* = 24)	29.50 (15.33)	31.50 (25.50)	*t*(49) = 0.51, *p* = 0.615
Vegan with lived eating disorder experience (*n* = 27)	27.44 (13.70)	27.00 (23.00)	
Anorexia nervosa (*n* = 24)	32.50 (11.75)	37.50 (24.25)	*t*(49) = 1.97, *p* = 0.055
Other diagnoses (*n* = 27)	24.78 (15.70)	26.00 (32.00)	

**Fig. 1:**
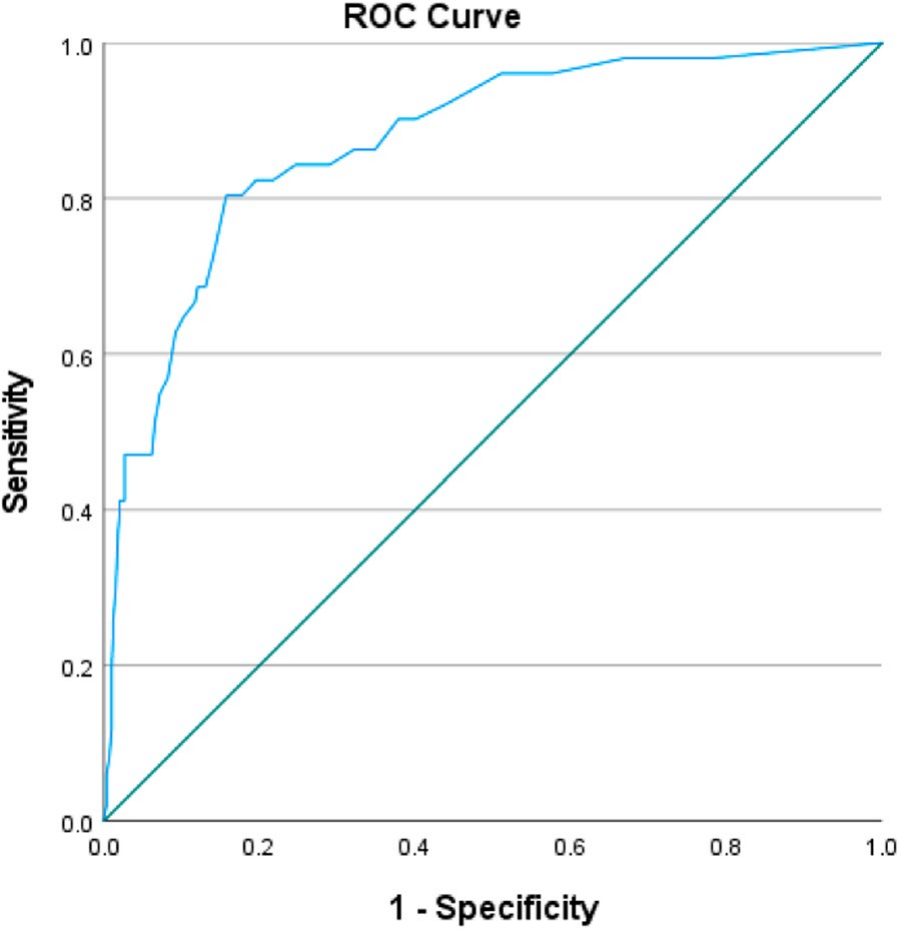
ROC curve analysis demonstrating the area under the curve for the prediction of eating disorder status using the V-EDS scored items

As shown in Fig. 1, ROC analysis indicates good discrimination power of the V-EDS score (AUC = 0.87, 95% confidence interval [CI] 0.82, 0.93). This indicates that 87% of the time a randomly selected eating disorder case would obtain a higher V-EDS score than a randomly selected non-clinical case.

According to Table 2, the cut-off score that provided the optimal trade-off between sensitivity and specificity as assessed by the Youden index was a global V-EDS score of ≥ 18, with 80.4% sensitivity and 84.3% specificity.

**Table 2:** Sensitivity, specificity, Youden index, and false positive rate for candidate cut-off scores for the V-EDS scored items

	V-EDS cut-off scores							
	≥ 12	≥ 13	≥ 14	≥ 15	≥ 16	≥ 17	≥ 18	≥ 19
Sensitivity	0.843	0.824	0.824	0.824	0.804	0.804	0.804	0.725
Specificity	0.754	0.782	0.796	0.804	0.822	0.831	0.843	0.859
Youden index	0.60	0.61	0.62	0.63	0.63	0.64	0.65	0.58
False positive rate	0.246	0.218	0.204	0.196	0.178	0.169	0.157	0.141

## Discussion

The present study fills an important gap as the first to investigate an optimal V-EDS score to discriminate the level of impairment from eating disorder pathology in a sample of vegetarian and vegan community and eating disorder participants. The V-EDS has been preliminarily established as a valid and reliable tool that can be used for initial screening and symptom progression of eating disorders in individuals adhering to a vegetarian or vegan diet [10]. We extend the utility of the V-EDS in discovering good performance of the V-EDS global score in predicting clinical caseness with a cut-off score of 18 shown to optimise the trade-off between sensitivity and specificity. We arrived at this cut-off score through the integration of the Youden index [18], a data-driven process to identify top-performing cut-offs.

These findings indicate that vegetarians and vegans at risk of eating disorders could potentially be initially screened through the administration of the V-EDS as a standalone measure in both clinical and research settings. The V-EDS could also be incorporated within a battery of other gold-standard measures, such as a clinical interview, for a comprehensive assessment and status confirmation. While we chose a clinical cut-off score that maximised both sensitivity and specificity with equal weight, users should consider the context and environment when employing the V-EDS for screening purposes. For example, a lower cut-off score could be used to enhance the sensitivity of the V-EDS (i.e., the true positive rate of respondents having an eating disorder and providing positive test results) and is considered an ideal quality for a “rule-out” test [19]. While this may result in the over-inclusion of non-cases, this could ensure the inclusion of individuals likely experiencing disordered eating and therefore could be a useful early intervention practice. The findings of this study suggest a V-EDS global score of 18 is optimal in predicting clinical caseness, however, a lower cut-off score could be incorporated if enhanced sensitivity is required.

A limitation of this study is that clinical eating disorder diagnosis was self-reported which meant that participants may not have identified as having an eating disorder if a formal diagnosis by a healthcare professional had not been provided. This also meant that our “clinical” sample may have included individuals who had not yet sought treatment for their eating disorder and may reflect the proportion of clinical participants who did not wish to self-classify their eating disorder diagnosis. Furthermore, our clinical sample was not large enough to be analysed by eating disorder diagnosis and therefore all clinical participants were grouped together. Indeed, of this sample, a large proportion of participants reported a diagnosis of anorexia nervosa (47%), which sits significantly above population prevalence rates within Australia [20]. While the prevalence of eating disorders in people who follow a vegetarian or vegan diet is very much a future research avenue to be explored using the V-EDS, it may be the case that people with a diagnosis of anorexia nervosa were interested in the present study due to the overlapping commonalities with the extensive dietary and lifestyle restrictions required to avoid animal-derived products. Future research could incorporate the use of structured clinical interview diagnoses and recruit larger groups of participants through treatment-seeking clinics to ensure participants meet a variety of diverse clinical diagnoses besides anorexia nervosa. Furthermore, the present sample was largely female-identifying and therefore generalisability of the findings to men, as well as gender-diverse people, is limited. We encourage future research to expand on developing the validity and reliability of the V-EDS in a range of diverse participant characteristic demographics including age, gender, sexuality, ethnicity, and eating disorder history. Last, as screening tools are invaluable sources of information to assist with decision-making in primary health settings [21], additional research is needed to explore the broad integration of the V-EDS within routine practice as a tool for continuous evaluation. Effective implementation of the V-EDS could potentially help ensure early assessment and treatment for minority groups such as vegetarians and vegans, in turn, reducing symptom worsening and long-term risks.

## Conclusions

In conclusion, this is the first study to investigate an optimal V-EDS score to discriminate the level of impairment from eating disorder pathology in a sample of vegetarian and vegan community and eating disorder participants. We found good discrimination power of the V-EDS, with evidence that supports employing a global cut-off score of 18 to predict clinical case status. Integration of the V-EDS into clinical practice may possibly be able to assist in improving eating disorder screening procedures for patients adhering to a vegetarian or vegan diet, in turn, aiding with the identification of those who may need further specialised eating disorder support. Further research should focus on expanding the psychometric properties of the V-EDS in diverse participant groups.

## Data Availability

The datasets generated and/or analysed from this study cannot be made available in line with ethics compliance.
